# Detrimental effects of simulated microgravity on mast cell homeostasis and function

**DOI:** 10.3389/fimmu.2022.1055531

**Published:** 2022-12-16

**Authors:** Minjin Kim, Gyeongin Jang, Kyu-Sung Kim, Jinwook Shin

**Affiliations:** ^1^ Inha Research Institute for Aerospace Medicine, Inha University College of Medicine, Incheon, Republic of Korea; ^2^ Department of Microbiology, Inha University College of Medicine, Incheon, Republic of Korea; ^3^ Department of Otorhinolaryngology-Head and Neck Surgery, Inha University Hospital, Incheon, Republic of Korea

**Keywords:** mast cells, microgravity, growth, survival, function

## Abstract

Exposure to microgravity causes significant alterations in astronauts’ immune systems during spaceflight; however, it is unknown whether microgravity affects mast cell homeostasis and activation. Here we show that microgravity negatively regulates the survival and effector function of mast cells. Murine bone marrow-derived mast cells (BMMCs) were cultured with IL-3 in a rotary cell culture system (RCCS) that generates a simulated microgravity (SMG) environment. BMMCs exposed to SMG showed enhanced apoptosis along with the downregulation of Bcl-2, and reduced proliferation compared to Earth’s gravity (1G) controls. The reduction in survival and proliferation caused by SMG exposure was recovered by stem cell factor. In addition, SMG impaired mast cell degranulation and cytokine secretion. BMMCs pre-exposed to SMG showed decreased release of β-hexosaminidase, interleukin-6 (IL-6), and tumor necrosis factor-α (TNF-α) upon stimulation with phorbol 12-myristate-13-acetate (PMA) plus calcium ionophore ionomycin, which correlated with decreased calcium influx. These findings provide new insights into microgravity-mediated alterations of mast cell phenotypes, contributing to the understanding of immune system dysfunction for further space medicine research.

## Introduction

Spaceflight studies have demonstrated that a harsh space environment induces dysregulation of the human immune system in complex ways (1, 2). Astronauts suffer from latent virus reactivation and diverse allergic reactions during shuttle and International Space Station flights (3–5). Microgravity is a key environmental factor responsible for immune dysfunction in astronauts during and after space missions (6, 7). Many ground-based instruments have been developed to simulate microgravity conditions, including rotating wall vessels (RWVs), clinostats, random positioning machines, and diamagnetic levitation (8). These tools allow us to investigate the effects of microgravity at the cell level cheaply but also to obtain results similar to those of space research (7, 9). Previous studies under SMG have reported various alterations in the growth, differentiation, signal transduction, function, and gene expression of hematopoietic stem cells (HSCs) and immune cells (10–19). However, the effects of microgravity on mast cells have not yet been documented.

Mast cells are tissue-resident immune cells of the hematopoietic lineage that participate in innate and adaptive immune responses against pathogens while also playing crucial roles in the pathogenesis of immediate hypersensitivity reactions and allergic diseases (20, 21). IL-3 and stem cell factor (SCF) are important for their maturation and survival (22). Activation of mast cells can be induced by the antigen crosslinking of immunoglobulin E (IgE) bound to FcεRI, SCF, compound 48/80, phorbol 12-myristate-13-acetate (PMA), and calcium ionophore (23). These immunological and chemical stimuli initiate multiple signaling events, including the phosphorylation of proximal protein tyrosine kinases and mitogen-activated protein kinases (MAPKs) as well as calcium immobilization, culminating in the immediate release of mediators stored in cytoplasmic granules such as histamine (24–26). In addition, the *de novo* synthesis of cytokines and chemokines in the activated cells contributes to late-phase allergic reactions including chronic inflammation (27, 28).

Herein, we explored whether microgravity affects mast cell homeostasis and function using murine bone marrow-derived mast cells (BMMCs) and a rotary cell culture system (RCCS) developed by the National Aeronautics and Space Administration (29) to generate an SMG culture environment. We showed that SMG negatively controls mast cell homeostasis by decreasing proliferation and increasing apoptosis. In addition, both degranulation and cytokine secretion were impaired in SMG-exposed cells after PMA plus calcium ionophore stimulation. Together, the proper gravitational force is a critical factor for the physiological and pathological roles of mast cells.

## Materials and methods

### BMMC culture

Bone marrow cells flushed from femurs and tibias of 6 to 8-week-old female C57BL/6 mice (DBL, South Korea) were cultured in IMDM−IL-3 medium for 4 to 5 weeks. IMDM−IL-3 is Iscove Modified Dulbecco Medium (IMDM; Welgene) supplemented with 10% fetal bovine serum (Hyclone), Penicillin-Streptomycin-Glutamine (Gibco), MEM Non-Essential Amino Acids (Gibco), 10 mM HEPES (pH 7.4), 1 mM sodium pyruvate, and 50 μM β-mercaptoethanol with 10% IL-3−conditioned medium generated from X63-IL-3 cells (kindly provided by Dr. X-P Zhong, Duke University Medical Center). BMMC purity was routinely monitored by flow cytometry using a BD FACS Calibur system (BD Biosciences) to detect FcεRI and c-Kit. BMMCs generated after culture for at least 4 weeks were used for further studies between 4 and 8 weeks during cell culture.

### SMG environment

**Figure 1:**
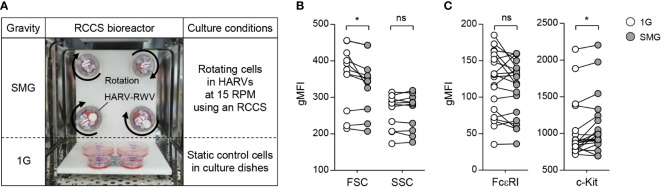
Effects of SMG on mast cell size, granularity, and surface expression of FcεRI and c-Kit. **(A)** BMMCs were cultured in HARV-RWVs of RCCS to simulate microgravity conditions or in static dishes under normal gravity (1G) controls. Round arrows indicate rotation. **(B)** Cell size and granularity were determined by geometric mean fluorescence intensity (gMFI) analyses of forward scatter (FSC) and side scatter (SSC) using flow cytometry after 48 h of SMG exposure, respectively (n = 11). **(C)** Scatter graphs represented surface expression of FcεRI and c-Kit on BMMCs after 48 h of SMG exposure. IgE-loaded cells were stained with PE-conjugated c-Kit and FITC-conjugated IgE antibodies (n = 20). Paired, two-tailed student’s t-test was performed to detect the statistical significance (*p < 0.05). ns, not significant.

In order to create an SMG culture environment on the ground, a rotary cell culture system (RCCS, Synthecon) consisting of a High Aspect Ratio Vessel (HARV)-RWV, rotator base, and power supply was used according to the manufacturer’s instruction. The HARV-RWV is a bioreactor with an oxygenator membrane that allows gas exchange. BMMC suspension (0.5×10^6^ cells/ml) in IL-3−conditioned medium with or without 100 ng/ml SCF (Peprotech) was injected into HARV-RWVs, followed by the removal of bubbles generated to minimize turbulence using sterile syringes. The vessels were attached to the rotator base set in a 37°C–5% CO_2_ incubator and rotated at 15 rpm for 24, 48, and 168 h (**Figure 1A**). The cells cultured in static dishes at the same time were used as a control.

### Flow cytometry

BMMCs (0.5×10^6^ cells/ml) were placed in HARV-RWVs of an RCCS for SMG or in static culture dishes for 1G and grown in a 37°C–5% CO_2_ incubator for 48 h. Thereafter, the cells were stained directly with phycoerythrin (PE)-conjugated anti-mouse c-Kit antibody (BioLegend) or stained at 1h after sensitization with 1 μg/ml IgE (Sigma-Aldrich) followed by incubation with fluorescein isothiocyanate (FITC)-conjugated anti-mouse IgE (BioLegend), and then analyzed by flow cytometry. For apoptosis analysis, cells cultured under SMG and 1G for 48 and 168 h were stained with Annexin V (A5)-PE (BioLegend) for 15 min at RT in a buffer containing 10 mM HEPES (pH 7.4), 140 mM NaCl, and 2.5 mM CaCl_2_. The proportions of A5-positive apoptotic cells were analyzed using a BD FACS Calibur system. To trace proliferation, cells were labeled with 5 μM CFSE (Invitrogen), a dye that split equally between the two daughter cells following each cell division, and exposed to SMG, SMG+1G, and 1G for 168 h. For SMG+1G, cells were cultured in HARV-RWV of RCCS, and after 24 h, the vessel was separated from RCCS, placed on the ground under 1G condition, and further incubated for 144 h. After the culture, the cells were stained with annexin V-PE. CFSE dilution in the A5-negative live population was analyzed. All the flow cytometry data were visualized using FlowJo V10 software (Tree Star).

### β-hexosaminidase and cytokine release

BMMCs (0.5×10^6^ cells/ml) exposed to either SMG or 1G for 24 and 48 h were harvested, washed 3 times with pre-warmed Tyrode’s solution (130 mM NaCl, 10 mM HEPES [pH 7.4], 1 mM MgCl_2_, 5 mM KCl, 1.4 mM CaCl_2_, 5.6 mM glucose and 1 mg/ml bovine serum albumin), and resuspended at 1×10^6^ cells/ml. 100 μl cells were seeded in a 96-well plate and treated with 100 μl of either 100 ng/ml phorbol 12-myristate-13-acetate (PMA, Sigma-Aldrich) plus 500 ng/ml ionomycin (Sigma-Aldrich) (P+I) or DMSO (Mock). At 15 min after P+I stimulation, the cell supernatants were incubated with p-nitrophenyl-N-acetyl-b-D-glucosamide (Sigma-Aldrich) as a β-hexosaminidase substrate for 30 min. The enzymatic reactions were terminated by the addition of NaOH. Absorbance at 405 nm with a reference filter at 620 nm was read using a Synergy HTX plate reader (BioTek). The total cellular β-hexosaminidase activity was quantified using supernatant lysed with 0.1% Triton X-100. The percentage of released β-hexosaminidase was analyzed by calculating the stimulatory activity relative to the total activity. For cytokine release analysis, BMMCs were grown under either SMG or 1G for 48 h, followed by resuspension at 1×10^6^ cells/ml and stimulation with P+I. The cell culture medium was collected at 6 h after stimulation and the amounts of IL-6 and TNF-α released were determined by ELISA assay using Mouse ELISA Max (BioLegend), according to the manufacturer’s protocol.

### Calcium flux

BMMCs exposed to either SMG or 1G for 48 h were loaded with FluoForte cell-permeable calcium binding dye using a FluoForte Ca^2+^ assay kit (Enzo Life Sciences, Farmingdale, NY, USA), according to the manufacturer’s protocol. Cells were resuspended at 0.5×10^6^ cells/ml in Hanks’ Buffer with 20 mM HEPES (HHBS) containing dye efflux inhibitor and seeded in 96-well black plate, followed by the addition of 250 ng/ml ionomycin and DMSO control. Calcium responses were visualized by recording fluorescence signals (485 nm excitation and 528 nm emission) using a Synergy HTX plate reader (BioTek) for 420 sec.

### Immunoblot assay

BMMC samples exposed to either SMG or 1G for 48 h were resuspended at 5×10^6^ cells/ml, stimulated with P+I for 10 min, washed twice with ice-cold phosphate-buffered saline (PBS), and lysed with radio-immunoprecipitation assay buffer (RIPA) supplemented with protease and phosphatase inhibitor cocktails (Sigma-Aldrich). The lysates were quantified using a Bradford protein assay kit (Bio-Rad) and protein samples were separated by SDS-PAGE, transferred onto Nitrocellulose membrane (Bio-Rad), and probed with the following primary antibodies: Bcl-2 (sc-7382) and Bax (sc-7480) from Santa Cruz Biotechnology, and Bcl-xL (#2764), Bax (#7480), BAK (#12105), Vinculin (#13901), phospho (p)-Erk1/2 (#4370), Erk1/2 (#4695), p-Jnk (#4668), Jnk (#9252), p-p38 (#4511) and p38 (#8690) from Cell Signaling Technology. All these antibodies were used at a 1:1000 dilution. Band densities in immunoblots were quantified by densitometry analysis using Adobe Photoshop CS6 (Adobe Systems Software).

### Statistical analysis

Data in graphs and immunoblot were presented as the mean ± standard error of the mean (SEM) and mean ± standard deviation (SD) of at least three independent experiments, respectively. Differences between data sets in graphs were evaluated by paired, two-tailed Student’s t-test using GraphPad Prism 5 software (GraphPad Prism Software Inc, San Diego, CA, USA) and considered statistically significant at p < 0.05 (* p < 0.05, ** p < 0.01, and *** p < 0.001).

## Results

### Effects of SMG on mast cell apoptosis and proliferation

**Figure 2:**
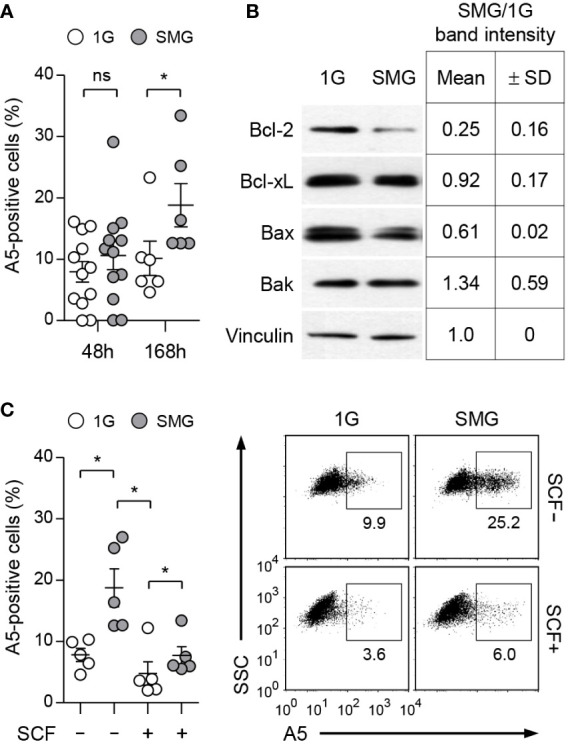
Effects of SMG on the apoptosis of mast cells. **(A)** BMMCs were cultured in IL-3 media at the indicated conditions. Apoptotic death was determined by analyzing Annexin V (A5)-positive stained cells using flow cytometry. Data shown are the mean ± SEM (n = 6 to 12). **(B)** Expression of Bcl-2 family proteins. Cells treated as in panel **(A)** for 48 h were lysed and analyzed by immunoblot with the indicated antibodies. Vinculin was used as the loading control. Band intensities were quantified by densitometry and the intensity ratios (SMG/1G) were shown as the mean ± SD (n = 3) from the samples of 3 independent experiments. **(C)** Cells were cultured with IL-3 ± 100 ng/ml SCF under SMG or 1G for 168 h and stained with PE-conjugated A5. Data shown are representative of 5 experiments (n = 5, mean ± SEM). *p < 0.05 by Student’s t-test. ns, not significant.

BMMCs were grown with IL-3 in RWVs of an RCCS bioreactor to create SMG and in static culture dishes for Earth’s gravity (1G) control (**Figure 1A**). As shown in **Figure 1B**, SMG-exposed cells showed comparable side scatter but reduced forward scatter compared to the control cells after 48 h of culture, indicating a decrease in cell size without affecting granularity by SMG. In addition, SMG exposure had no effect on FcεRI expression but increased c-Kit expression on the cell surface (**Figure 1C**). Elevated apoptotic death and reduced proliferation of T lymphocytes have been reported under both real microgravity and SMG conditions (30–33). To determine whether microgravity affects mast cell apoptosis, the cells were stained with fluorochrome-conjugated Annexin V (A5). There was no obvious difference in the apoptotic ratios between these cells after 48 h of culture; however, it increased by 1.9-fold in SMG-exposed cells after 168 h compared to 1G control cells (**Figure 2A**), suggesting that mast cells are more sensitive to apoptosis under SMG. To further investigate the molecular mechanism by which SMG promotes apoptosis in mast cells, we analyzed the protein levels of pro-survival Bcl-2 and Bcl-xL and pro-apoptotic Bax and Bak using immunoblot analysis. The Bcl-2 levels in SMG-exposed cells were significantly lower than those of 1G control cells (**Figure 2B**). The expression ratios of Bcl-2, Bcl-xL, Bax, and Bak relative to 1G controls in SMG-exposed cells were calculated to be 0.25, 0.92, 0.61, and 1.34, respectively. The c-Kit ligand stem cell factor (SCF) functions as a growth and survival factor in mast cells (34). To investigate whether SCF affects SMG-induced apoptosis in mast cells, BMMCs were cultured in IL-3 or IL-3+SCF medium in HARV-RWVs for 168 h. SMG-exposed cells exhibited increased apoptosis with or without SCF but survived in SCF medium at rates comparable to 1G controls (**Figure 2C**), indicating that SCF rescues mast cells from SMG-induced apoptosis.

**Figure 3:**
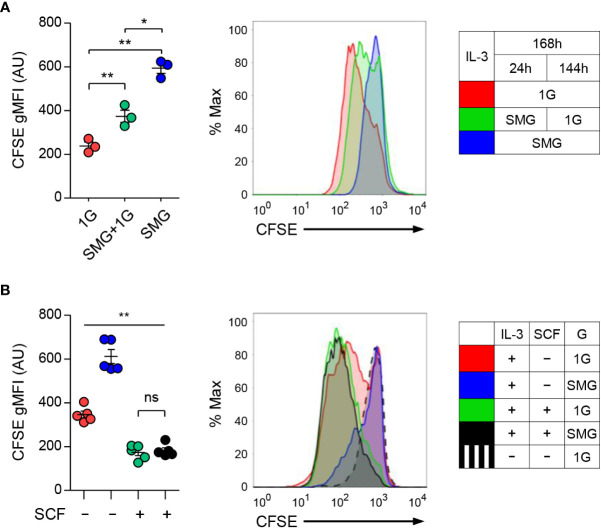
Effects of SMG on the proliferation of mast cells. **(A)** Carboxyfluorescein succinimidyl ester (CFSE)-labeled BMMCs were cultured with IL-3 under 1G, SMG+1G, and SMG conditions for 168 h. CFSE is a cell-permeable dye diluted during cell division. After the exclusion of A5-stained cells, CFSE dilutions were monitored using flow cytometry. Data are shown as the mean ± SEM (n = 3). **(B)** CFSE-labeled cells were cultured with IL-3 ± SCF under SMG or 1G for 168 h and cell proliferation was analyzed using flow cytometry. Data are shown as the mean ± SEM of 5 experiments. The gMFI of each graph represents the cell proliferation activity. CFSE-labeled cells cultured without IL-3 and SCF were used as a negative control for cell proliferation (black dashed line). The P-value comparisons between all groups in **(B)** were determined as **p < 0.01 except ns, not significant. *p < 0.05.

To investigate whether microgravity affects mast cell growth, we labeled BMMCs with CFSE dye used as an indicator of cell proliferation and cultured them under 1G, SMG+1G, and SMG conditions. As shown in **Figure 3A**, the levels of CFSE were higher as exposure time to SMG increased, indicating the decline of cell proliferation under SMG, which was restored by SCF treatment (**Figure 3B**). Together, these results demonstrate that SMG negatively regulates mast cell homeostasis by inducing apoptosis and suppressing proliferation.

### Effects of SMG on mast cell effector functions

**Figure 4:**
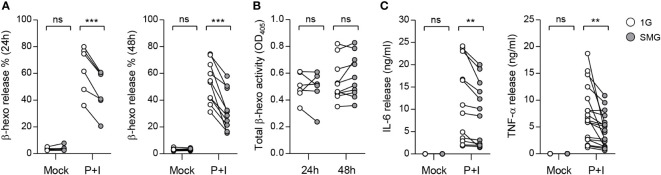
Effects of SMG on mast cell functions. BMMCs were exposed to SMG for 24 to 48 h, and then incubated in Tyrode’s solution with either 50 ng/ml phorbol 12-myristate-13-acetate (PMA) plus 250 ng/ml ionomycin (P+I) or DMSO (Mock). **(A)** Degranulation was accessed by measuring β-hexosaminidase release at 15 min after stimulation. Pairwise scatter plots represented the percentages of released β-hexosaminidase (n = 6 at 24 h; n = 10 at 48 h). **(B)** Total β-hexosaminidase activity in **(A)** was measured using a cell supernatant dissolved in Triton X-100 and represented by a scatter graph. **(C)** Cells were exposed to SMG for 48 h and the amounts of IL-6 and TNF-α cytokines in the culture media were determined using ELISA at 6 h after stimulation (n = 12 in IL-6; n = 18 in TNF-α). **p < 0.01 and ***p < 0.001, Student’s t-test. ns, not significant.

Activation of mast cells results in the degranulation of preformed mediators and the release of newly synthesized cytokines in a phasic manner (35). To investigate the effects of SMG on mast cell effector functions, we stimulated BMMCs that were cultured under SMG for 48 h with the diacylglycerol analog PMA plus calcium ionophore ionomycin (P+I). Co-treatment of diacylglycerol analog and calcium ionophore can mimic the FcεRI-mediated activation of mast cells (36, 37). Following activation, β-hexosaminidase released together with histamine from mast cell granules is widely used as an indicator of degranulation (38, 39). Cellular functional degranulation was assessed by measuring the enzymatic activity of β-hexosaminidase on its substrate. The P+I-induced release of β-hexosaminidase was significantly diminished by SMG pre-exposure (**Figure 4A**), indicating the inhibition of P+I-induced degranulation by SMG. The activities in whole cell lysates were not obviously different between these cells (**Figure 4B**), indicating the minimal effect of SMG on β-hexosaminidase production. In addition, to evaluate the effect of SMG on the late-phase function of activated mast cells, the culture media were collected at 6 h after stimulation and were subjected to ELISA to measure secreted cytokines. Compared to 1G controls, the amounts of IL-6 and TNF-α secreted from SMG-pre-exposed cells were decreased by 20% and 36%, respectively (**Figure 4C**). Together, these results demonstrated that SMG inhibits both immediate and late-phase reactions in mast cell activation.

### Effects of SMG on calcium flux and MAPK signals

**Figure 5:**
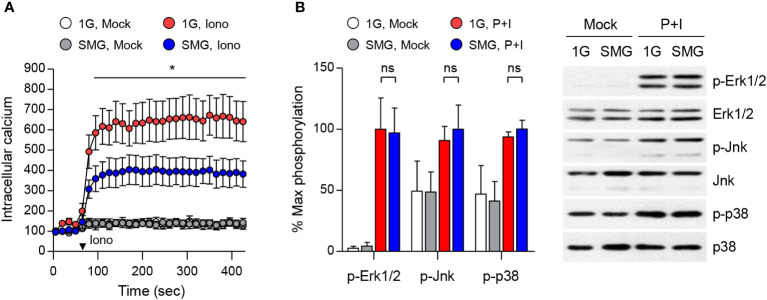
Effects of SMG on calcium influx and MAPK signaling. **(A)** After exposure to either 1G or SMG, BMMCs loaded with FluoForte calcium binding dye were treated with 250 ng/ml ionomycin (Iono) at the time marked by an inverted triangle. Intracellular calcium levels were visualized by fluorescence microplate reader for 420 sec. Data are shown as the mean ± SEM of 5 independent experiments. **(B)** BMMCs exposed to either 1G or SMG were stimulated with P+I for 10 min and the cell lysates were subject to immunoblot assay using the indicated antibodies. Band intensities of p-Erk1/2, p-Jnk, and p-p38 were normalized to their total protein expression and are shown as the mean ± SEM (n = 8 in p-Erk1/2; n = 3 in p-Jnk; n = 3 in p-p38). *p < 0.05 by Student’s t-test. ns, not significant.

To understand the mechanism of mast cell dysfunction under SMG, we examined calcium mobilization in mast cells. Calcium signal acts as a second messenger in mast cells and is essential for degranulation and the production and release of cytokines including IL-6 and TNF-α (40, 41). BMMCs exposed to either SMG or 1G were loaded with FluoForte calcium binding indicator followed by ionomycin treatment. As shown in **Figure 5A**, ionomycin-triggered calcium influx was significantly reduced by SMG exposure, which suggests that SMG impairs mast cell effector function by inhibiting the calcium signal. MAPKs also play critical roles in mast cell activation (36, 42). The phosphorylations of Erk1/2, Jnk, and p38 MAPKs were induced by P+I treatment, but no obvious differences were detected between the cells exposed to SMG and 1G (**Figure 5B**).

## Discussion

During deep-space exploration, astronauts are exposed to harsh environmental factors such as microgravity, ionizing radiation, and extreme temperatures, all of which reportedly induce a series of changes in human physiology, including vision impairment, bone and muscle loss, and nervous and immune system dysregulation (43, 44). Among these, immune dysfunction has been particularly concerning because of its clinical impacts on astronauts during and after space missions. A growing body of studies has reported the impacts of spaceflight on both innate and adaptive immune cells. Examples include altered activation and gene expression profiles of T cells (45–47), decreased phagocytosis and oxidative burst capacity of monocytes and neutrophils (48, 49), and decreased cytotoxicity of natural killer cells (50). Recent case studies have documented the incidence of persistent skin rash, hypersensitivity, and rhinitis onboard the International Space Station (4, 5). Since the results of spaceflight research are complexly affected by various space environments, it is difficult to interpret the effects of specific factors. This present study provides the first evidence that microgravity is an environmental inhibitor for mast cell survival and function.

The balance and interaction between pro-survival and pro-apoptotic Bcl-2 family decide the cell life or death. SMG-induced apoptosis with a reduction in Bcl-2 has been reported in endothelial cells (51, 52), osteoblastic cells (53, 54), and carcinoma cells (55). Bcl-2 can bind to activated Bax, thereby preventing it from disrupting the mitochondrial outer membrane (56). We found that exposure to SMG enhanced apoptosis but reduced the proliferation of mast cells, when cultured in IL-3 medium (**Figures 2A**, **3A**). Correlated with increased apoptosis, diminished expression of Bcl-2 was observed in these cells, where the ratios of Bax/Bcl-2 protein levels increased to about 2.4 (**Figure 2B**), suggesting that microgravity controls mast cell apoptosis through the mitochondrial pathway. Interestingly, the upregulation of microRNAs targeting *bcl-2* messages under SMG has been reported. Following RWV culture, the upregulation of miR-503-5p induced endothelial cell apoptosis by decreasing Bcl-2 (52) and the increased level of miR-34a was detected in lymphoblastoid cells (57). Thus, the increase of unknown microRNAs may be associated with diminished Bcl-2 expression and enhanced apoptosis in SMG-exposed mast cells in the current study. Furthermore, we found that SCF supplement efficiently recovered the defects in survival and proliferation (**Figures 2C**, **3B**). Given that Bcl-2 is a key regulator of apoptosis in mast cells (58–60), and its expression is upregulated by SCF (59, 61), this supports the notion that unbalancing the Bcl-2 family, mainly by blocking Bcl-2, contributes to SMG-induced apoptosis in mast cells. The recovery of proliferation by SCF indicates that SCF signaling is intact in mast cells under SMG. In contrast, SMG reportedly inhibits the proliferation of HSCs by decreasing the SCF-Kit-Ras/cAMP-CREB pathway (62). In addition, short-term exposure to SMG caused a more severe reduction in the Concanavalin A-induced proliferation of CD4T cells than CD8T cells (31). Together, these results suggest that the action of microgravity depends on cell types and cell contexts. The molecular mechanism by which SMG controls mast cell survival and proliferation remains to be characterized.

Ca^2+^ signaling plays a central role in various cell lineages, including lymphocytes. Studies on lymphocytes have documented that short-duration spaceflight reduced IL-6 production in human blood samples upon P+I stimulation (63), and SMG pre-exposure decreased IL-2 and IFN-γ production in T cells upon P+I stimulation (13, 31). We showed that SMG-pre-exposed BMMCs manifest a decline in degranulation as well as IL-6 and TNF-α secretion in response to P+I stimulation (**Figure 4**), which correlated with decreased Ca^2+^ responses to ionomycin (**Figure 5A**). Thus, it is possible that microgravity contributes to immune dysfunction through altered Ca^2+^ regulation. Intracellular Ca^2+^ mobilization is a critical process in granule exocytosis and cytokine production of mast cells (41); it also plays important roles in mast cell motility, chemotaxis, and the synthesis of arachidonic acid, which is the precursor for leukotrienes and prostaglandins. Thus, it is also possible that microgravity suppresses not only degranulation and cytokine production but also other Ca^2+^-related functions in mast cells. A recent study showed that SMG suppressed macrophage development from HSCs by altering the Ras/Erk/NF-κB pathway (16). This study also showed that IFN-γ and lipopolysaccharide-induced TNF-α and IL-6 productions were reduced in macrophages differentiated under SMG. However, there were no obvious differences in P+I-induced Erk1/2, Jnk, and p38 phosphorylation in our study (**Figure 5B**), suggesting that microgravity desensitizes mast cells to P+I stimuli through MAPK-independent pathways.

In summary, microgravity is a negative regulator of mast cell homeostasis and function. SMG inhibits mast cell growth and survival through the downregulation of Bcl-2. In addition, it impaired the P+I-induced degranulation and cytokine secretion, which were associated with impaired Ca^2+^ influx. The effects of microgravity on mast cells require further studies to determine its physiological relevance. However, our findings provide new insights into microgravity-induced immune dysfunction and suggest the application of microgravity environments on Earth as a potential therapeutic strategy in mast cell-related diseases.

## Data availability statement

The raw data supporting the conclusions of this article will be made available by the authors, without undue reservation.

## Ethics statement

The animal study was reviewed and approved by the Institutional Animal Care and Use Committee of Inha University (INHA 161214-465).

## Author contributions

K-SK and JS contributed conception and design of the study. MK and GJ performed the experiments. MK, GJ, JS analyzed the data. JS wrote the first draft of the manuscript. All authors contributed to manuscript revision, read and approved the submitted version.
